# Caspase-1 activation, IL-1/IL-6 signature and IFNγ-induced chemokines in lungs of COVID-19 patients

**DOI:** 10.3389/fimmu.2024.1493306

**Published:** 2025-01-15

**Authors:** Audrey Cambon, Christophe Guervilly, Clémence Delteil, Nicola Potere, Richard Bachelier, Edwige Tellier, Evelyne Abdili, Marine Leprince, Marco Giani, Ildo Polidoro, Valentina Albanese, Paolo Ferrante, Laurence Coffin, Michael Schiffrin, Laurent Arnaud, Romaric Lacroix, Sandrine Roque, Jean-Marie Forel, Sami Hraiech, Laurent Daniel, Laurent Papazian, Françoise Dignat-George, Gilles Kaplanski

**Affiliations:** ^1^ Aix-Marseille Université, INSERM, INRAE, C2VN, Marseille, France; ^2^ Centre d’Etudes et de Recherches sur les Services de Santé et qualité de vie EA 3279, Aix-Marseille Université, Marseille, France; ^3^ Service de Médecine Intensive Réanimation, Hôpital Nord, Assistance Publique- Hôpitaux de Marseille, Chemin des Bourrely, Marseille, France; ^4^ Département de Médecine légale, Hôpital de la Timone, Assistance Publique-Hôpitaux de Marseille, Marseille University, Marseille, France; ^5^ School of Medicine and Health Sciences, “G. d’Annunzio” University of Chieti-Pescara, Chieti, Italy; ^6^ Service d’Hématologie et de Biologie vasculaire, CHU La Timone, APHM, Marseille, France; ^7^ Service de Médecine interne et d’Immunologie clinique, Assistance Publique - Hôpitaux de Marseille, Hôpital La Conception, Marseille, France; ^8^ Unit of Legal Medicine, “Santo Spirito” Hospital, Local Health Authority of Pescara, Pescara, Italy; ^9^ AB2 Bio, Lausanne, Switzerland; ^10^ Service d’Anatomopathologie, APHM, Aix Marseille University, Marseille, France; ^11^ Service de Réanimation, Centre Hospitalier de Bastia, Bastia, France

**Keywords:** acute respiratory distress syndrome, vasculopathy, caspase-1, cytokines, bronchoalveolar fluid, COVID-19

## Abstract

**Rationale:**

COVID-19-associated acute-respiratory distress syndrome (C-ARDS) results from a direct viral injury associated with host excessive innate immune response mainly affecting the lungs. However, cytokine profile in the lung compartment of C-ARDS patients has not been widely studied, nor compared to non-COVID related ARDS (NC-ARDS).

**Objectives:**

To evaluate caspase-1 activation, IL-1 signature, and other inflammatory cytokine pathways associated with tissue damage using post-mortem lung tissues, bronchoalveolar lavage fluids (BALF), and serum across the spectrum of COVID-19 severity.

**Methods:**

Histological features were described and activated-caspase-1 labeling was performed in 40 post-mortem biopsies. Inflammatory cytokines were quantified in BALF and serum from 19 steroid-treated-C-ARDSand compared to 19 NC-ARDS. Cytokine concentrations were also measured in serum from 128 COVID-19 patients at different severity stages.

**Measurements and main results:**

Typical “diffuse alveolar damage” in lung biopsies were associated with activated caspase-1 expression and vascular lesions. Soluble Caspase-1p20, IL-1β, IL-1Ra, IL-6 and at lower level IFNγ and CXCL-10, were highly elevated in BALF from steroid-treated-C-ARDS as well as in NC-ARDS. IL-1β appeared concentrated in BALF, whereas circulating IL-6 and IL-1Ra concentrations were comparable to those in BALF and correlated with severity. TNFα, TNFR1 and CXCL8 however, were significantly higher in NC-ARDS compared to C-ARDS, treated by steroid.

**Conclusions:**

In the lungs of C-ARDS, both caspase-1 activation with a predominant IL-1β/IL-6 signature and IFNγ -associated chemokines are elevated despite steroid treatment. These pathways may be specifically targeted in ARDS to improve response to treatment and to limit alveolar and vascular lung damage.

## Highlights

Caspase-1 activation and a predominant IL-1/IL-6 signature associated with IFNγ-induced chemokines remain highly detectable in the BALF of steroid-resistant COVID-19-ARDS, arguing for new multi-targeted therapeutics in COVID-19-ARDS. Lung biopsies from deceased COVID-19 patients show Diffuse Alveolar Damage and vasculopathy associated with activated-caspase-1. In the lungs of C-ARDS and NC-ARDS, a predominant caspase-1-induced IL-1β/IL-6 signature and IFNγ -induced chemokines persist.

## Introduction

Coronavirus-disease-2019 (COVID-19), caused by severe-acute-respiratory-syndrome-coronavirus-2 (SARS-CoV-2) is associated with dysregulation of host immune responses (1). In severe forms, COVID-19 causes bilateral pneumonia, that rapidly progresses to acute-respiratory-distress-syndrome (ARDS) and death (1). Immunothrombosis is one of the main complications arising in severe forms of COVID-19, due to the combined hyperactivation of the immune and coagulation systems (2–5). Multiple immune pathways have been shown to play a role in the immunopathogenesis of COVID-19. Type I interferons (IFN) promote the activation of antiviral effector mechanisms and induce the production of pro-inflammatory cytokines (6, 7). Conversely, the impaired type III-IFN response observed in severe cases of COVID-19 may promote innate immunity with hyperinflammation and immunothrombosis (2, 8–10). Increased circulating levels of pro-inflammatory cytokines have been detected in patients with severe COVID-19, as well as increased activation of nuclear-factor-kappa-light chain enhancer of activated B cells (NF-kB) (11). Inflammasomes markers such as the NACHT, LRR, pyrin domain (PYD) domains-containing protein 3 (NLRP3) and the absent-in-melanoma-2 (AIM2) are also activated during COVID-19 (12). Danger-associated molecular patterns (DAMPs) and pathogen-associated molecular patterns (PAMPS) released upon tissue injury are detected by NLRP3, a cytoplasmic protein which is in turn, activated into a macromolecular complex called NLRP3-inflammasome (13, 14). The activated NLRP3-inflammasome engages through the PYD domain of ASC (apoptosis-associated speck-like protein containing a caspase recruitment domain [CARD]) pro-caspase-1, which then undergoes autocatalytic activation to caspase-1. Activated caspase-1 (Casp1p20) is then responsible for transforming pro-IL-1β and pro-IL-18 into biologically active IL-1β and IL-18, two major pro-inflammatory and pro-coagulant cytokines of the IL-1 family. Casp1p20 also cleaves gasdermin-D, which forms cytoplasmic membrane pores and triggers pyroptosis (15). During COVID-19, strong activation of the NLRP3-inflammasome and caspase-1 is induced with subsequent release of IL-1β and IL-18, although these cytokines remain difficult to detect in patient’s blood (14, 16). Although controversial, caspase-1-induced pyroptotic cell death also appears to occur following SARS-CoV-2 infection of mononuclear cells *in vitro* (15, 17–19). Furthermore, in humanized mice expressing the human ACE2 receptor, SARS-CoV-2 infects macrophages by binding of anti-spike IgG to CD16, resulting in NLRP3-inflammasome activation, IL-1β and IL-18 production, and pyroptosis (19–21). Both pyroptosis and necrosis play an important role in hyperinflammatory syndromes, mainly through a significant release of DAMPs, notably IL-1α, which maintains an upward inflammatory loop (20, 22–24). IL-1α induces inflammasome activation and IL-1β production and, like IL-1β, is mediated through the IL-1 receptor. Despite their central role in amplifying the innate immune response, the role of DAMPs in COVID-19 is poorly understood to date. Moreover, the specific contribution of NLRP3 inflammasome activation and IL-1 signaling to COVID-19 immunopathogenesis remains unclear. In the present study of three prospective cohorts of COVID-19-patients, we investigated caspase-1 activation and IL-1 signature using post-mortem lung tissue, bronchoalveolar lavage fluid (BALF), and serum across the spectrum of COVID-19 severity. We also investigated the hypothetical role of other important inflammatory pathways and DAMPs in COVID-19 immunopathogenesis.

## Methods

### Patients characteristics and samples

This prospective multicenter study was approved by the Medical Ethics Committee of Aix-Marseille-University (CPP # 1123 HPS1) and by the Assistance Publique de Marseille digital data protection delegate (RGPD2020-47). Patients were divided into three different cohorts (**Supplementary Figure S1**). All patients underwent a nasopharyngeal swab to confirm SARS-CoV-2 infection with RT-PCR.

Cohort A, consisted of post-mortem lung samples from 40 patients admitted to the public hospitals of Marseille (France) and “Santo Spirito” hospital in Pescara (Italy) with ARDS and SARS-CoV-2 infection who died of respiratory failure. For comparison, we used lung samples from 10 patients with ARDS and a negative RT-PCR SARS-CoV-2, who died from other causes than COVID-19: drowning, thoracic traumatism, epiglottitis, bladder perforation, aortic dissection, congenital cardiopathy and unexplained death.

Cohort B, consisted of 38 patients enrolled between July 1st, 2019 and April 23rd, 2021 in two tertiary university extracorporeal membrane oxygenation (ECMO) centers in Marseille (Hôpital Nord) and Paris (Pitié-Salpétrière). We included intubated and mechanically ventilated (IMV) adults with severe ARDS receiving veno-venous-ECMO for less than 24 hours. 19 patients were RT-PCR confirmed SARS-CoV-2 infected (C-ARDS) and 19 patients were COVID-19 free, with negative RT-PCR for SARS-CoV-2 (NC-ARDS). All C-ARDS patients received dexamethasone 6 mg intravenously for 10 days as standard treatment according to the RECOVERY protocol (17) and anticoagulation. All ARDS-patients underwent BAL and blood sampling within 48 hours of ECMO cannulation and baseline characteristics as well as clinical outcomes were recorded. BALF and serum were collected and stored at -80°C prior to analysis. BALF from 12 patients diagnosed with lung cancer were used as controls. We chose to use these controls because these patients were in diagnostic phasis. All patients had early-stage lung cancer and early-stage lung cancer is a disease with very low systemic and bronchoalveolar inflammation. Of these, 9 patients had non-small-cell lung cancer and 3 patients had small-cell lung cancer.

Cohort C, consisted of 128 patients with COVID-19 pneumonia confirmed by positive RT-PCRand lung computed tomodensitometry, admitted to public hospitals of Marseille, between March 20th, 2020 and April 14th, 2020. Patients were classified according to their clinical manifestations and severity, like in the protocol for novel coronavirus pneumonia (25). 37 patients were hospitalized in intensive care unit with critical COVID-19 and required invasive mechanical ventilation (IMV), were categorized as IMV-COVID-19 patients, (n=37); 21 patients were hospitalized in medical units with severe COVID-19 and required supplemental O_2_ >6L/min to achieve peripheral oxygen saturation (SpO_2_) ≥95% (severe COVID-19, n=21), 70 patients were hospitalized in medical units with COVID-19 not meeting the criteria for severe or IMV-COVID-19 and therefore categorized as moderate COVID-19 (n=70). Baseline clinical data and clinical outcomes were recorded for all patients. Serum was collected within 48 hours of hospitalization and stored at -80°C. Serum was also collected from 11 healthy volunteers COVID-19 free, with a negative RT-PCR for SARS-CoV2.

### Post-mortem lung tissue sampling and characterization

The autopsies were performed by experienced forensic pathologists in accordance with published recommendations (26). The organs were studied both *in situ* and individually on the dissection table. Lungs were biopsied transparietally (pleura and lung parenchyma samples), in the posterior region and inferior lobar. Pathologic examination was performed on each organ fixed in 10% buffered formalin. Microscopic examinations of the lungs were performed on the central and peripheral areas of each inferior lobe, using 4µm sections stained with hematoxylin, eosin and saffron (HES). The most representative lung samples were analyzed by immunohistochemistry. Slides were incubated overnight at 4°C with a rabbit polyclonal antibody (Ab) anti-activated-caspase-1 (1:100; Merck, St-Louis, USA), followed by an secondary rabbit antibody and HRP-DAB. Images were acquired with the NanoZoomer S360 (Hamamatsu, Massy, France) using a 10×objective (×100 magnification) for HES and for immunohistochemistry.

### Cytokine assays

Soluble Caspase-1 p20 (sCasp1p20) and human IL-1 receptor antagonist (IL-Ra) concentrations were evaluated by QUANTIKINE ELISA assays (Bio-Techne Minneapolis, USA), IL-1α by ProQuantum Human IL-1α immunoassay (Thermo Fisher Scientific, Waltham, USA), HMGB1 by ELISA kit (Bio-Techne), IL-33 by ELISA kit (Abcam, Cambridge, UK), IL-1β by human IL-1β High-Sensitivity ELISA kit (Thermo Fisher Scientific) and soluble sNLRP3, C-X-C motif chemokine ligand (CXCL)10, IL-6, and IL-18 using specific ELISA assays (BD Biosciences, San Jose, USA). IFNγ concentration was quantified by Luminex kit (Thermo Fisher Scientific), and tumor necrosis α (TNFα), soluble tumor necrosis factor receptor-1 (sTNFR-1) and CXCL8 by Luminex kits from Merck (St-Louis, USA).

### Statistical analysis

GraphPad-Prism V.9.2.0 software (GraphPad Software Inc., San Diego, USA) was used for statistical analysis. Values are presented as median with interquartile range (25%-75% percentile) for the indicated number of dosages. Comparisons between groups were performed using Mann-Whitney test for quantitative variables. Before carrying out these statistical tests, we performed a one-factor ANOVA test to highlight any significant differences between the groups. The ANOVA test with a p<0.05 value prompted us to perform a Mann-Whitney test, comparing the groups 2 by 2.

Associations between continuous variables were analysed using Spearman-correlation-test. Statistical significance was defined as p<0.05.

## Results

### Caspase-1 activation is mostly associated with vasculopathy in lungs with C-ARDS

**Figure 1:**
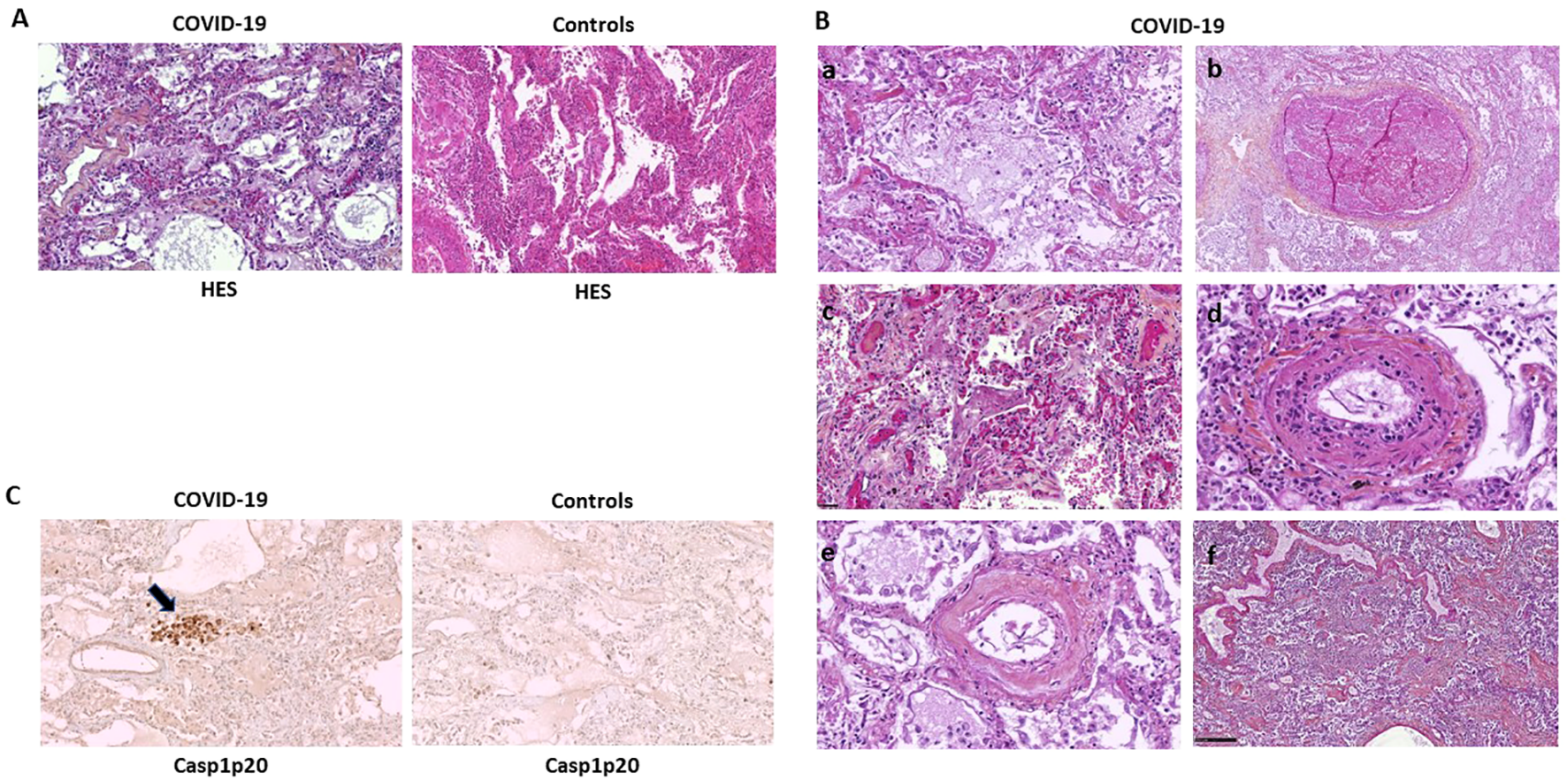
Caspase-1 activation is mostly associated with vascular lesions in C-ARDS lung samples. Paraffine-embedded lung sections (4µm) from patients who died of COVID-19 (**A**, left panel and **B**) or controls (**A**, right panel) stained by hematoxylin-eosin-saffron (HES) coloration (original magnification x100). Microscopic aspect of lesions such as Diffuse Alveolar Damage (DAD) with desquamation of alveolar epithelium (**A**, left panel and **B**a), thrombosis (**B**b), intra-alveolar fibrin deposits (**B**c), endothelitis (**B**d and **B**e) and interstitial infiltration by mononuclear inflammatory cells (**B**f). Activated Caspase-1 (Casp1p20) expression in lung sections of patients who died of COVID-19 (**C**, left panel) or controls (**C**, right panel), was analyzed by anti-Casp1p20 immunostaining of macrophages and desquamative pneumocytes (original magnification x100). Scale bar = 250µm.

We first examined pathologic findings and *in situ* caspase-1 activation in C-ARDS-related lung injury using postmortem lung samples from 40 deaths (**Table 1**). Typical features of alveolar injury (**Figure 1A**, left panel) were observed in 72.5% of cases among which 23 had diffuse alveolar damage (DAD) (**Figure 1B**c). Evidence of vasculopathy was present in 34 cases distributed as follows: 8/34 with thrombo-embolisms (**Figure 1B**b), 8/34 with thrombotic microangiopathy (**Figure 1B**c), 6/34 with endothelitis (**Figure 1B**d) and 26/34 with intimal lesions (**Figures 1B**d, **B**e). In 20/40 of the cases, interstitial inflammation was present, mainly consisting in the presence of mononuclear cells in inflammatory lesions. We then performed immunohistochemistry on lung sections using specific anti-Casp1p20 Ab (**Figure 1C**). Caspase-1 activation was detected in 14 of 29 (48.2%) lung biopsies presenting with alveolar lesions, in 10/23 (43.4%) of those presenting DAD (**Figure 1C**, left panel), but in only in 1/10 (10%) among controls. Moreover, Casp1p20 labelling was mostly associated with endoalveolar macrophages (15/20) rather than with interstitial inflammatory cells (4/20) or pneumocytes (1/20). Casp1p20 labelling associated with vascular lesions in 90% of cases (18/20), with alveolar lesions in 70% (14/20) and with interstitial lesions in 45% (9/20).

**Table 1:** Histological analysis of lung sections from 40 patients who died of COVID-19 ARDS: frequency of alveolar, interstitial, vascular lesions and Casp1p20 expression.

TOTAL LUNG SAMPLE	40
**Alveolar+Interstitial+Vascular lesions**	**14/40 (35%)**
**Alveolar lesions**	**29/40 (72.5%)**
Casp1p20 +	*14/29*
*DAD*	*23/29*
DAD Casp1p20 +	*10/23*
**Interstitial inflammation**	**20/40 (50%)**
Casp1p20 +	9/20
**Vascular lesions**	**34/40 (85%)**
Casp1p20 +	18/34
*Isolated TE*	8/34
*Isolated TMA*	*8/34*
*TE and TMA*	*1/34*
***Thrombotic lesions***	***17/34 (50%)***
*Endothelitis*	*6/34*
*Endothelitis+Intimal lesions*	*10/34*
*Intimal fibrosis*	*14/34*
*Intimal edema*	*6/34*
*Intimal edema+ fibrosis*	*6/34*
***Inflammatory lesions***	***26/34 (76.4%)***
***Thrombotic + inflammatory lesions***	***14/34 (41.1%)***
**Total Casp1p20 positive biopsies**	**20/40 (50%)**
*Casp1p20+ endoalveolar macrophages*	*15/20 (75%)*
*Casp1p20+ other inflammatory cells*	*4/20*
*Casp1p20+ pneumocytes* *Casp1p20+ vascular lesions* *Casp1p20+ alveolar lesions* *Casp1p20+ interstitial lesions*	*1/20* *18/20 (90%)* *14/20 (70%)* *9/20 (45%)*

DAD, Diffuse Alveolar Damage; TE, Thromboembolism; TMA, Thrombotic microangiopathy. The bold/italicized values mean number/total (percent).

### Elevated sNLRP3 and caspase-1 levels in BALF and serum of COVID-19 patients

**Figure 2:**
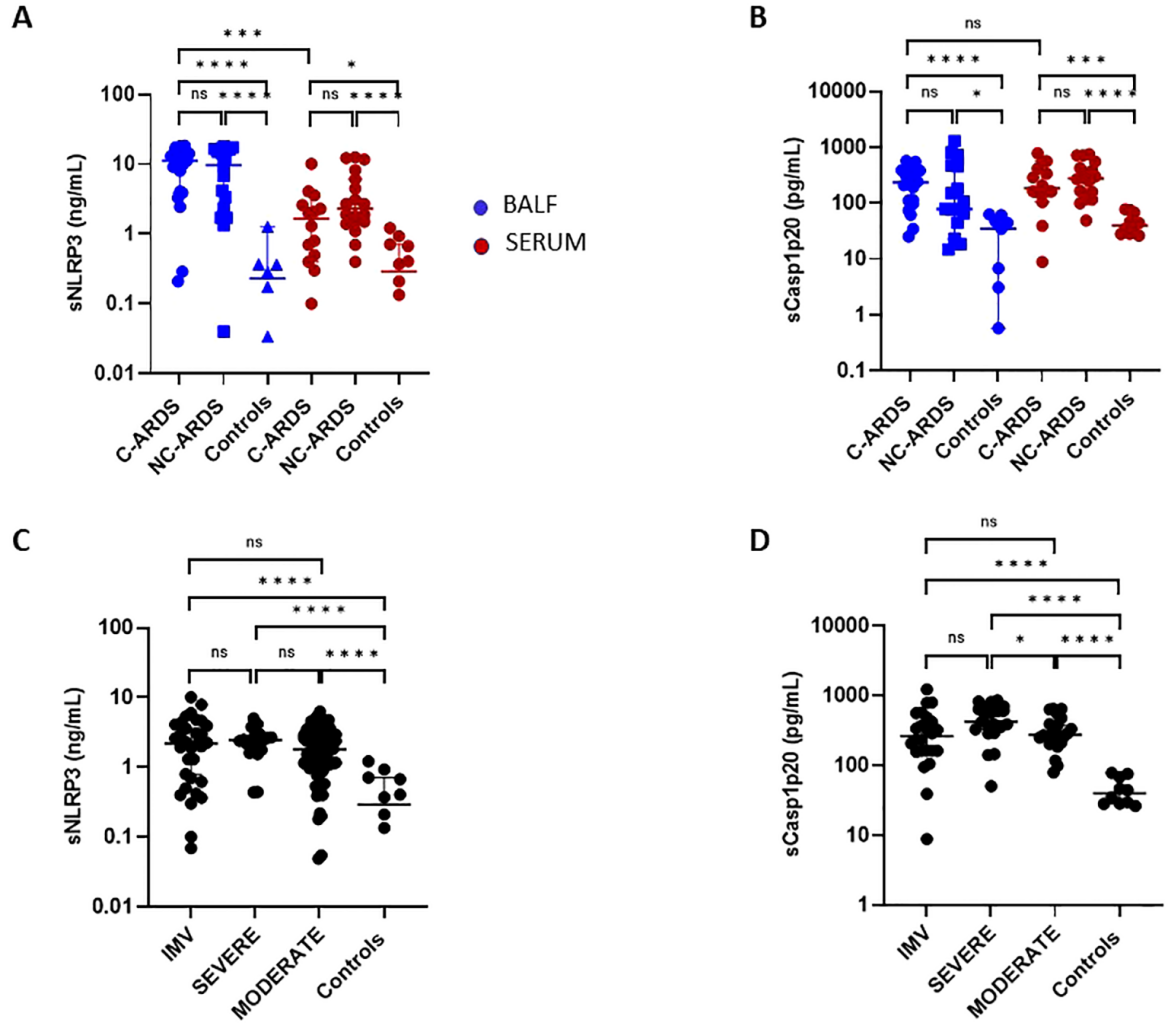
Elevated sNLRP3 and sCasp1p20 concentrations in C-ARDS and in NC-ARDS. sNLRP3 **(A)** and sCasp1p20 **(B)** proteins concentrations were measured by ELISA in the BALF (blue) from patients with COVID-19 ARDS (C-ARDS, n=19), non-COVID-19 ARDS (NC-ARDS, n=19) or with lung cancer as respective controls (Controls, n=8). Concentrations of the same proteins were measured in the serum (red) from patients with C-ARDS (n=14), NC-ARDS (n=19) or healthy donors as respective controls (n=12). sNLRP3 **(C)** proteins concentrations were measured in the serum from COVID-19 patients (**C**: n=122) and sCasp1p20 **(D)** proteins concentrations were measured in the serum from COVID-19 patients (**D**: n=69) and from healthy donors as respective controls (n=10). COVID patients were classified in IMV forms (**C**: n=32 and **D**: 24 respectively), severe forms (**C** n= 20 and **D**: n=21 respectively) and moderate forms (**C**: n= 70 and **D**: 24 respectively) and compared to controls. Numbers of patients tested for cytokines assays could varied in each cohort, according to technical difficulties. Each dot represents the value from a single individual (*p<0.05, ***p<0.001, ****p<0.0001). ns, no statistically significant.

First, we examined the activation of the NLRP3-inflammasome signaling pathway. sNLRP3 was significantly increased in BALF from C-ARDS and NC-ARDS patients compared to controls (p<0.0001, respectively; **Figure 2A**). Similar results were obtained when sCasp1p20 was measured in BALF (**Figure 2B**). sNLRP3 was also significantly increased in the serum of C-ARDS and NC-ARDS patients compared to controls (p<0.05 and p<0.0001 respectively; **Figure 2A**), as was sCasp1p20 in the serum of C-ARDS and NC-ARDS patients compared to controls (p<0.001 and p<0.0001, respectively; **Figure 2B**). However, no significant difference was found, when comparing sNLRP3 and sCasp1p20 levels in BALF from C-ARDS versus NC-ARDS patients (p=0.66 and p=0.16, respectively; **Figures 2A, B**). Importantly, sNLRP3 levels were significantly higher in BALF from C-ARDS patients compared to serum from the same patients (p<0.001; **Figure 2A**), while this was not true for sCasp1p20 (p>0.05, **Figure 2B**).

We then assessed circulating sNLRP3 and sCasp1p20 levels in relation to disease severity (**Figures 2C, D**). Overall, serum sNLRP3 levels were significantly increased in COVID-19 patients compared to controls at all disease stages (**Figure 2C**; **Table 2**). Similar results were obtained for sCasp1p20 levels (**Figure 2D**; **Table 2**). When stratified, sCasp1p20 levels were significantly higher in patients with severe versus moderate COVID-19 (p<0.01; **Figure 2D**).

**Table 2:** Associations of cytokines and pro-inflammatory proteins with COVID-19 severity.

	PLASMA										
	Median concentration (pg/ml) [CI]				p values						
	(a) IMV	(b) SSevere	(c) Moderate	(d) Healthy Donors	(a) vs (d)	(b) vs (d)	(c) vs (d)	(a) vs (b)	(a) vs (c)	(b) vs (c)	(a)+(b)+(c) vs (d)
**IL-1β**	0.278 [0.128-0.908]	0.4150 [0.186-0.77]	0.166 [0.1-0.297]	0.004 [0.00-0.005]	p<0.0001	p<0.0001	p<0.0001	p<0.01	p<0.01	p>0.05	p<0.0001
**IL-1Ra**	2446[1567-4792]	2021[1227-7128]	1726 [592.1-4482]	240.2 [202.6-397.5]	p<0.0001	p<0.0001	p<0.0001	p=0.066	p>0.05	p>0.05	p<0.0001
**IL-6**	62.32 [34.39-147.1]	24.08 [13.46-67.38]	25 [14.68-46.51]	0.38 [0.00-2.50]	p<0.0001	p<0.0001	p<0.0001	p<0.001	p<0.0001	p>0.05	p<0.0001
**IL-18**	478.5 [404.8-798.3]	801.5 [553.5-1283]	590.5 [438.3-715]	394 [316-475]	p<0.001	p<0.0001	p<0.001	p<0.05	p>0.05	p<0.05	p<0.001
**CXCL10**	214.3 [125.1-339.5]	2146 [74.87-407.3]	106.4 [53.85-209.6]	15.14 [11.63-19.51]	p<0.0001	p<0.0001	p<0.0001	p<0.01	p<0.0001	p<0.01	p<0.0001
**NLRP3** **(ng/ml)**	2.17[0.64-4.06]	2.46[1.74-2.90]	1.79[1.01-2.93]	0.29[0.00-0.70]	p<0.0001	p<0.0001	p<0.0001	p>0.05	p>0.05	p>0.05	p<0.0001
**Casp1p20**	261.1 [161.1-468.6]	421.4 [309.4-666.7]	273.3 [216.9-386.0]	39.58 [28.27-69.75]	p<0.0001	p<0.0001	p<0.0001	p>0.05	p>0.05	p<0.01	p<0.0001

### Activation of the IL-1β/ IL-6 pathway in C-ARDS

**Figure 3:**
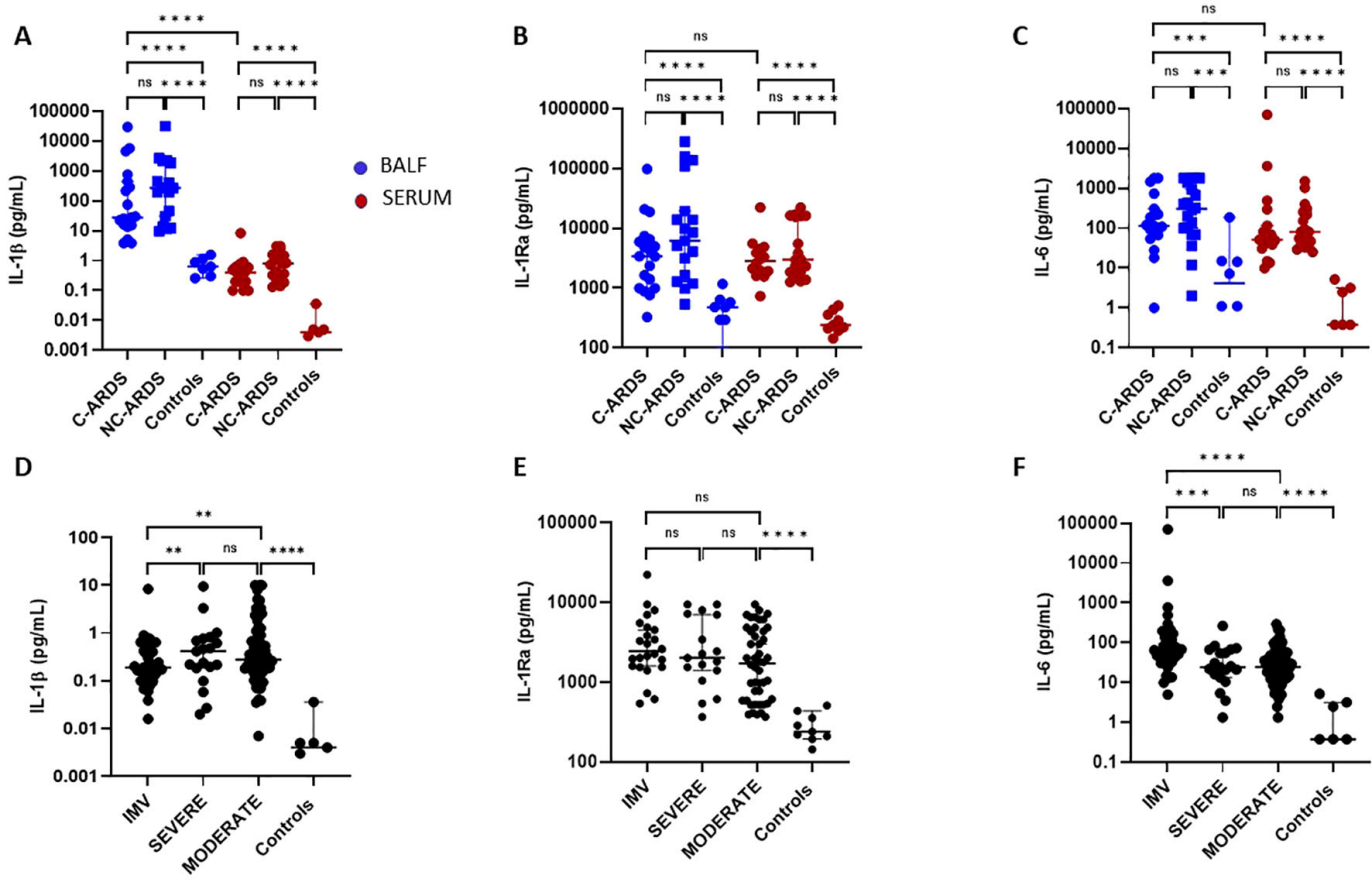
Activation of the IL-1β/IL-6 pathway in C-ARDS and in NC-ARDS. IL-1β **(A)**, IL-1Ra **(B)**, and IL-6 **(C)** concentrations were measured in the BALF (blue) from patients with C-ARDS (n=19), NC-ARDS (n=19) or with lung cancer as specific controls (Controls, n=8). Concentrations of the same proteins were measured in serum (red) from patients with C-ARDS (n=19), NC-ARDS (n=19) or healthy donors as specific controls (n=9). IL-1β **(D)**, IL-1Ra **(E)**, and IL-6 **(F)** concentrations were measured in the serum from COVID-19 patients (n=128) and healthy donors as respective controls (n=8). COVID patients were classified in IMV (**D**: n=37, **E**: n=24 and **F**: n=37 respectively), severe (**D**: n= 21, **E**: n=17 and **F**: n=21 respectively) and moderate forms (**D**: n =70, **E**: n= 46 and **F**: n=70 respectively) and compared to controls. Numbers of patients tested for cytokines assays could varied in each cohort, according to technical difficulties. Each dot represents the value from a single individual (ns: p>0.05, **p<0.01, ***p<0.001, ****p<0.0001).

To assess the consequences of NLRP3-inflammasome and caspase-1 activation in COVID-19 patients, we measured the levels of IL-1β, IL-1Ra and IL-6 in both BALF and serum of C-ARDS or NC-ARDS patients and in control subjects. Despite inter-individual variability, IL-1β (p<0.0001 and p<0.0001, respectively; **Figure 3A**), IL-1Ra (p<0.0001 and p<0.0001, respectively; **Figure 3B**) and IL6 (p<0.001 and p<0.001 respectively; **Figure 3C**) concentrations were significantly increased in BALF from C-ARDS and NC-ARDS patients compared to controls. In serum, IL-1β was detectable at very low concentrations but was significantly higher in C-ARDS and NC-ARDS patients compared to controls (p<0.0001 and p<0.0001, respectively; **Figure 3A**). In contrast, the concentrations of IL-1Ra (p<0.0001 and p<0.0001, respectively; **Figure 3B**) and IL-6 concentrations (p<0.0001 and p<0.0001, respectively; **Figures 3C**) were well detectable in the serum and significantly higher in C-ARDS and NC-ARDS than in controls. We also evaluated whether cytokine concentrations in BALF and serum differed between C-ARDS and NC-ARDS. We found no significant differences in IL-1β, IL-Ra and IL-6 concentrations in subjects with C-ARDS versus NC-ARDS in either BALF or serum (**Figures 3A–C**). Interestingly, IL-1β levels, from subjects with C-ARDS, were significantly higher in BALF than in serum (p<0.0001; **Figure 3A**), unlike IL-1Ra and IL-6 (p>0.05 and p>0.05; respectively; **Figures 3B, C**).

We then assessed serum IL-1β, IL-1Ra and IL-6 concentrations in COVID-19 patients according to disease severity. Serum IL-1β was increased in all COVID-19 patients compared to healthy subjects (**Table 2**, **Figure 3D**) as was IL-1Ra. (**Table 2**, **Figure 3E**), and IL-6 (**Table 2**, **Figure 3F**). Intriguingly). Interestingly, circulating IL-1β levels were lower in patients with IMV-COVID-19 compared to severe or moderate COVID-19 (**Table 2**, **Figure 3D**), while no difference was observed for IL-1Ra (p>0.05; **Figure 3E**). Moreover, circulating IL-6 levels were significantly higher in patients with IMV-COVID compared to severe or moderate COVID-19 (**Table 2**, **Figure 3F**).

### Activation of the IL-18/IFNγ pathway in C-ARDS

**Figure 4:**
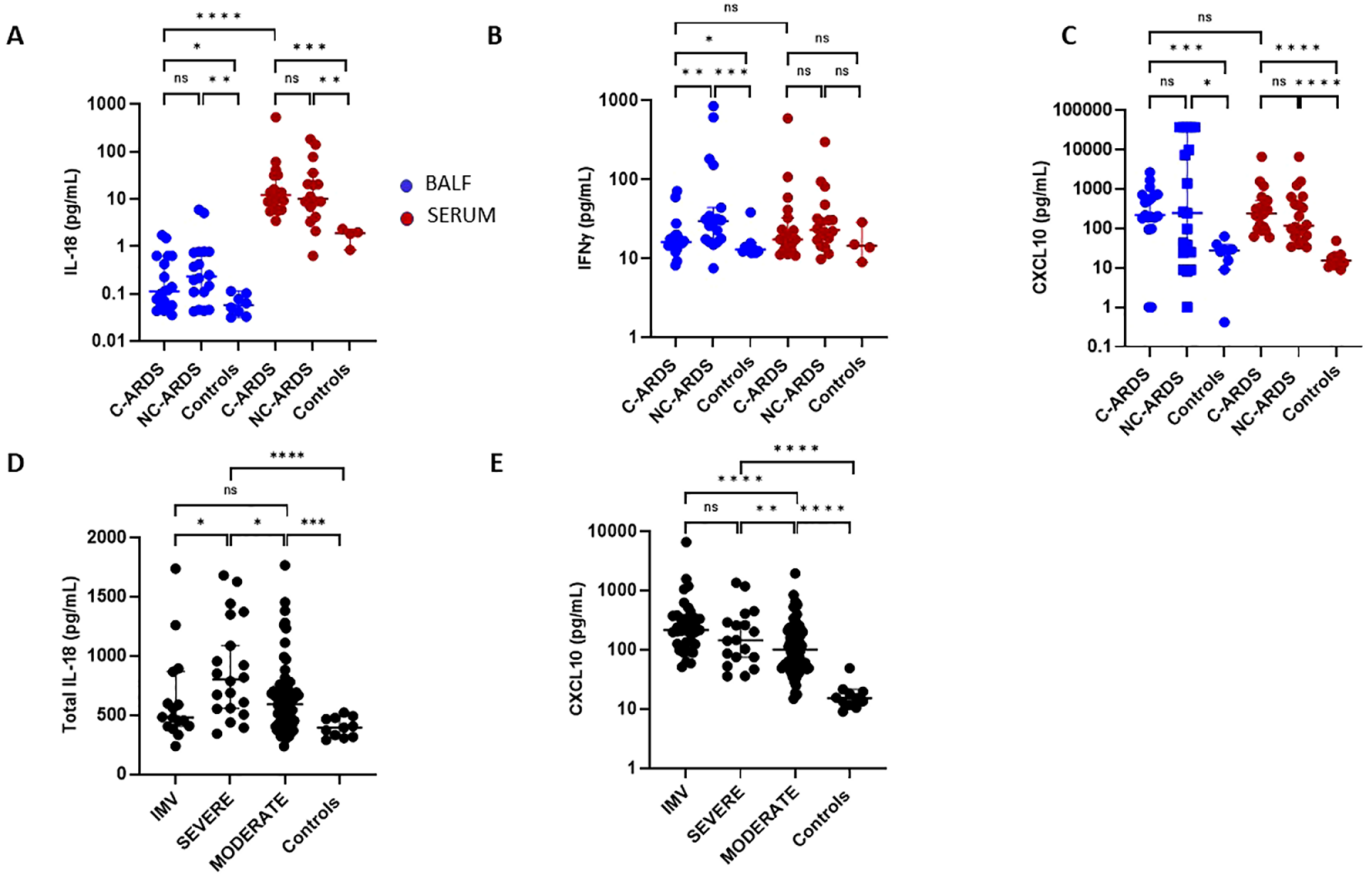
Activation of the IL-18/IFNγ pathway in C-ARDS and in NC-ARDS. IL-18 **(A)**, IFNγ **(B)** and CXCL10 **(C)** concentrations were measured in the BALF (blue) from patients with C-ARDS (n=19), NC-ARDS (n=18) and with lung cancer as specific controls (n=8). Concentrations of the same proteins were measured in serum (red) from patients with C-ARDS, (n=19), NC-ARDS, (n=19) and healthy donors as controls (**A**: n=4, **B**: n= 4 and **C**: n=11 respectively). IL-18 **(D)** and CXCL10 **(E)** concentrations were measured in serum from COVID-19 patients (n= 127) and from healthy donors as respective controls (n=11). COVID patients were classified in IMV (**D**: n=16 and **E**: n= 37, respectively), severe (n=20) and moderate forms (n= 70) and compared to controls. Numbers of patients tested for cytokines assays could varied in each cohort, according to technical difficulties. Each dot represents the value from a single individual (*p<0.05, **p<0.01, ***p<0.001, ****p<0.0001). ns, no statistically significant.

Since production of IL-18/IFNγ can also be affected by NLRP3-inflammasome and caspase-1 activation, we assessed IL-18, IFNγ, and CXCL10 levels in BALF and serum of patients with C-ARDS or NC-ARDS and of control subjects. In BALF, IL-18 was elevated in both C-ARDS and NC-ARDS patients compared to the controls (p<0.05 and p<0.01, respectively; **Figure 4A**) but detectable at very low concentrations. IL-18 was considerably higher in serum than in BALF from C-ARDS patients (p<0.0001, **Figure 4A**) and in serum of C-ARDS and NC-ARDS patients than in controls (p<0.001 and p<0.01 respectively; **Figure 4A**).

IFNγ levels in BALF were significantly higher in both C-ARDS and NC-ARDS than in controls (p<0.05 and p<0.001, respectively; **Figure 4B**). Conversely, serum INFγ concentrations from C-ARDS and NC-ARDS patients were not different from controls (p>0.05 respectively; **Figure 4B**). CXCL10 was increased in BALF from C-ARDS and NC-ARDS compared to controls (p<0.001 and p<0.05, respectively; **Figure 4C**) and in serum from C-ARDS and NC-ARDS compared to controls (p<0.0001 and p<0.0001, respectively; **Figure 4C**). However, no significant differences of CXCL10 concentrations were observed when comparing BALF from C-ARDS versus NC-ARDS (p=0.98; **Figure 4C**).

When stratifying according to disease severity, serum IL-18 was overall increased in all COVID-19 subgroups compared to controls (**Table 2**, **Figure 4D**). Circulating IL-18 was slightly higher in severe compared to moderate COVID-19 (p<0.05; **Figure 4D**), but lower in IMV-COVID-19 compared to severe COVID-19 (p<0.05; **Figure 4D**). Serum CXCL10 was increased in all COVID-19 patients compared to controls, (**Table 2**, **Figure 4E**). Plasma). Serum levels of CXCL10 appeared to increase with the severity of COVID-19 (**Table 2**, **Figure 4E**).

To define predictive biomarkers of severity, we then explored the correlation between serum cytokine levels and widely used scores reflective of patient clinical status. Circulating IL-1Ra and IL-6 positively correlated with SOFA score (p<0.01, respectively; **Supplementary Figures S2A, B**), whereas sNLRP3 concentrations positively correlated with SAPS2 score (p<0.05; **Supplementary Figure S2C**).

### Elevated DAMPS in BALF from C-ARDS

**Figure 5:**
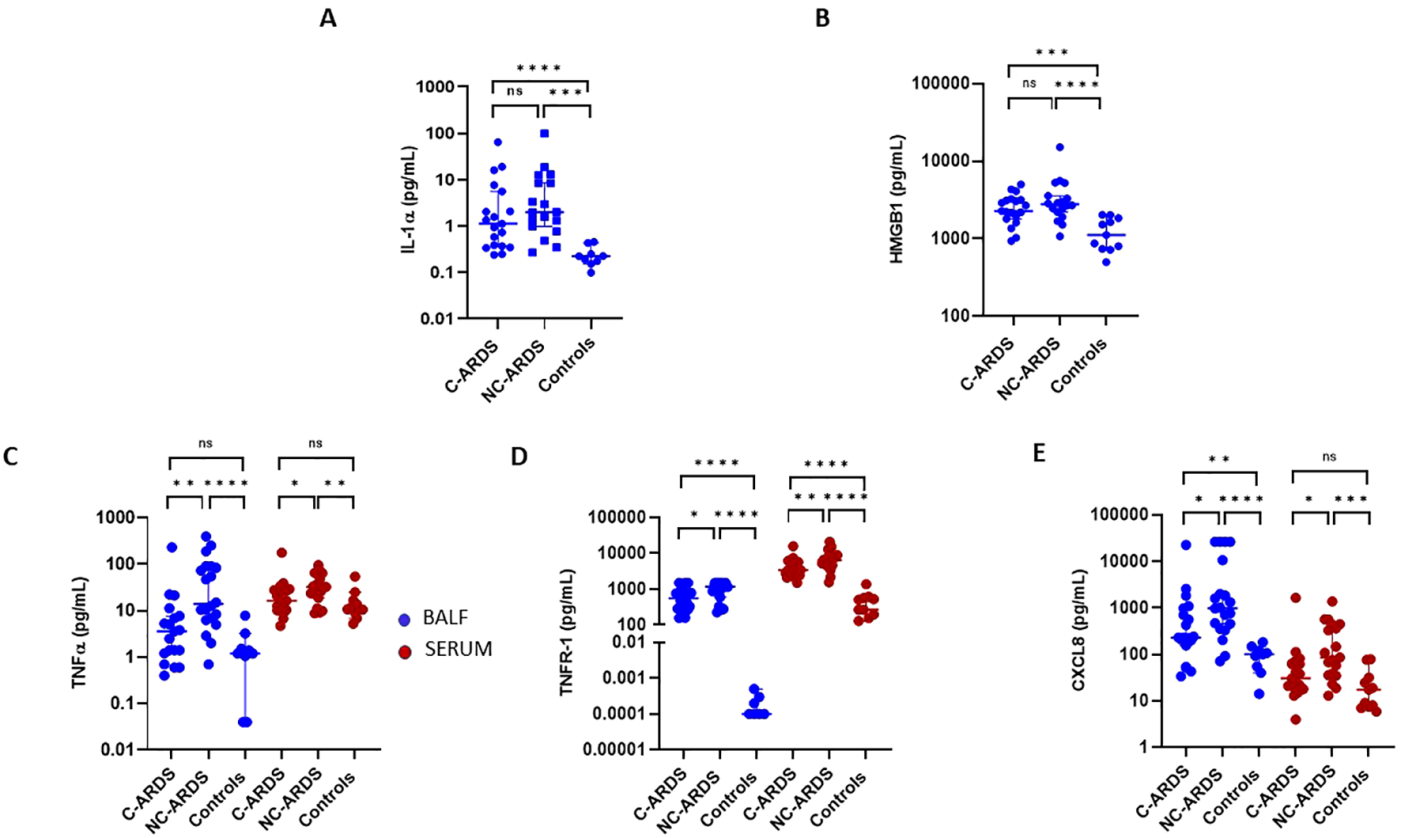
Elevated DAMPS concentrations in BALF of C-ARDS, and TNFα, sTNFR-1, CXCL8 concentrations in C-ARDS and NC-ARDS. IL-1α **(A)** and HMGB1 **(B)** concentrations were measured in the BALF from patients with C-ARDS (n=19), NC-ARDS (n=18) and with lung cancer as respective controls (n=11). TNFα **(C)**, TNFR-1 **(D)**, CXCL8 **(E)** concentrations were measured in the BALF (blue) from patients with C-ARDS (n=19), NC-ARDS, (n=19) and with lung cancer as controls (n=8). Concentrations of the same proteins were measured in serum (red) from patients C-ARDS, NC-ARDS and healthy donors as controls (n=10). Numbers of patients tested for cytokines assays could varied in each cohort, according to technical difficulties. Each dot represents the value from a single individual (*p<0.05, **p<0.01, ***p<0.001, ****p<0.0001). ns, no statistically significant.

To evaluate the pathogenic contribution of DAMPs in C-ARDS, we measured IL-1α and HMGB1 levels in BALF of C-ARDS or NC-ARDS patients and of control subjects. Both IL-1α and HMGB1 levels were detectable and significantly higher in BALF from C-ARDS and NC-ARDS patients than from controls (for IL-1α, p<0.0001 and p<0.001, respectively; **Figure 5A** and for HMGB1, p<0.001 and p<0.0001, respectively; **Figure 5B**). However, no difference was observed between patients with C-ARDS and with NC-ARDS concerning IL-1α or HMGB1 concentrations in BALF (p=0.20 and p=0.3, respectively; **Figures 5A, B**).

### Lower TNFα, sTNFR-1 and CXCL8 concentrations in C-ARDS

To determine the contribution of other inflammatory cytokines to the immunopathogenesis of C-ARDS, we measured TNFα, TNF-R1 and CXCL8 in BALF and serum of patients with C-ARDS, NC-ARDS and of controls. Surprisingly, concentrations of TNFα, sTNFR-1 and CXCL8 in BALF of C-ARDS were significantly lower than in NC-ARDS (**Table 3**, **Figures 5C–E**), but significantly higher than in controls for sTNFR-1 and CXCL8 (**Table 3**, **Figures 5D, E**). Similarly, serum levels of TNFα, sTNFR-1 and CXCL8 in C-ARDS were significantly lower than in NC-ARDS (**Table 3**, **Figures 5C–E**) and serum levels of sTNFR-1 was higher than in controls (p<0.0001; **Figure 5D**).

**Table 3:** Cytokine concentrations in BALF and plasma from COVID-19/non-COVID-19 ARDS patients and respective controls.

	BALF						PLASMA					
	Median concentration (pg/ml) [CI]			p values			Median concentration (pg/ml) [CI]			p values		
	(a) C-ARDS	(b) NC-ARDS	(c) Cancer controls	(a) vs (c)	(b) vs (c)	(a) vs (b)	(d) C-ARDS	(e) NC-ARDS	(f) Healthy donors	(d) vs (f)	(e) vs (f)	(d) vs (e)
**IL-1α**	1.130 [0.37-5.58]	2 [0.94-9.51]	0.224 [0.16-0.34]	p<0.0001	p<0.0001	p>0.05	–	–	–	–	–	–
**HMGB-1**	2288 [1808-3178]	2785 [2131-4002]	1119 [738.1-1850]	p<0.001	p<0.0001	p>0.05	–	–	–	–	–	–
**NLRP3** **(ng/ml)**	11210 [3.91-15.94]	9630 [1.84-16.37]	230 [0.008-0.37]	p<0.0001	p<0.0001	p>0.05	1650 [470-2850]	2850 [1470-6620]	290 [0.00-700]	p<0.05	p<0.0001	p>0.05
**Casp1p20**	234 [97.26-379,8]	78.43 [21.79-458.8]	34.59 [1.83-53.99]	p<0.0001	p<0.05	p>0.05	184.6 [143.5-452.6]	275.3 [122.9-455.3]	39.58 [28.27-69.75]	p<0.001	p<0.0001	p>0.05
**IL-1β**	27.55 [15.43-447.6]	270.3 [22.39-2028]	0.6390 [0.3048-1.165]	p<0.0001	p<0.0001	p>0.05	0.4 [0.20-0.60]	0.80 [0.326-1.59]	0.004 [0.000-0.005]	p<0.0001	p<0.0001	p=0.07
**IL-1Ra**	3404 [995.6-6518]	6213 [1283-19384]	478.4 [146.8-606.1]	p<0.0001	p<0.0001	p>0.05	1666 [956.8-3817]	3001 [1754-16 000]	240.2 [202.6-397.5]	p<0.0001	p<0.0001	p>0.05
**IL-6**	114.0 [67.00-307.0]	303.0 [77.0-1275]	4.15 [0.27-14.88]	p<0.001	p<0.001	p>0.05	51.83 [38.55-114.9]	81.02 [40.36-254.3]	0.38 [0.00-2.50]	p<0.0001	p<0.0001	p>0.05
**IL-18**	0.1129 [0.053-0.63]	0.23 [0.09-0.77]	0.057 [0.035-0.09]	p<0.05	p<0.01	p>0.05	12.22 [6.83-31.40]	10.11 [4.85-31.80]	1.91 [1.09-2.25]	p<0.01	p<0.001	p>0.05
**IFNγ**	16.14 [14.53-19.76]	29.67 [17.15-71.13]	12.98 [012.14-14.43]	p<0.05	p<0.001	p<0.01	17.48 [12.78-32.64]	23.00 [16.41-36.08]	14.46 [10.22-25.37]	p>0.05	p>0.05	p>0.05
**IP10**	218 [181-711]	171.0 [12.75-30219]	27.29 [12.17-34.26]	p<0.001	p>0.05	p>0.05	239.0 [98.21-6509.4]	116.2 [63.27-571.4]	15.14 [11.63-48.74]	p<0.0001	p<0.0001	p>0.05
**TNFα**	3.6 [1.20-7.70]	13.90 [6.45-88.88]	1.19 [0.54-2.38]	p=0.07	p<0.0001	p<0.01	16.4 [10.3-27.8]	32.5 [19.0-64.1]	11.02 [8.98-16.55]	p>0.05	p<0.01	p<0.05
**TNFR-1**	554.0 [285.0-800.0	1186 [598.0-1500]	0.000 [0.0001-0.0003]	p<0.0001	p<0.0001	p<0.05	3402.0 [2577.0-5775.0]	6452 [4586-8868]	266.7 [156.0-570.5]	p<0.0001	p<0.0001	p<0.01
**CXCL8**	232.0 [156.0-987.0]	996.0 [382.5-8460]	101.3 [48.10-134.5]	p=0.0051	p<0.0001	p<0.05	31.0 [18.00-64.00]	86 [36- 442]	17.49 [7.51-31.81]	p=0.08	p<0.001	p<0.05

## Discussion

In this prospective multicenter study, we used several approaches such as histopathological and immunohistochemical analyses on post-mortem tissues from subjects with fatal C-ARDS, and immune profiling of BALF and serum from patients across the COVID-19 severity spectrum to provide information on NLRP3-inflammasome activation and the involvement of IL-1 signaling in COVID-19 patients.

Consistent with previous reports (27–30), DAD was present in almost 60% of subjects with fatal COVID-19-ARDS, and interstitial mononuclear cell inflammation in half of them. Inflammatory vascular lesions, including endothelitis, were detected in the vast majority (>80%) of subjects died of COVID-19 disease. In the same time, pulmonary thromboembolism and thrombotic microangiopathy were also frequent (>40%), confirming the idea that vasculopathy, coagulopathy and immunothrombosis contribute centrally to the pathogenesis of refractory ARDS in patients with COVID-19 (2, 29, 31–34).

Expression and localization of Casp1p20, an indicator of NLRP3-inflammasome activation, were assessed in the lungs of patients with refractory C-ARDS. Interestingly, intense Casp1p20 expression was also detected in abundance (>90%) at sites of vascular injury and vessel thrombosis, indicating a spatial association between NLRP3-inflammasome activation and COVID-19-associated vasculopathy.

In agreement with autopsy findings, increased levels of sNLRP3 and sCasp1p20 were observed in both BALF and serum from C-ARDS, with sNLRP3 concentrations considerably higher in BALF than in serum. The lung is thus a major site of inflammasome assembly and activation during C-ARDS. In addition, serum levels of sNLRP3 and sCasp1p20 were globally increased in all COVID-19 patients, with Casp1p20 concentrations higher in severely ill compared to moderate ill patients. This suggests the involvement of caspase-1 in hyperinflammatory responses. Collectively, these findings are consistent with activation of the NLRP3-inflammasome pathway in both lungs and blood and also corroborate previous observations showing elevated Casp1p20 levels in COVID-19 patients and inflammasome activation in patient-derived PBMCs and in CD14^+^ macrophages resident in COVID-19 lung tissue (17, 29, 35, 36). NLRP3-inflammasome activation can be directly triggered by multiple SARS-CoV-2-derived proteins, including spike protein and ORF3a, which act as PAMPs (19).

Activation of the NLRP3-inflammasome and subsequent cleavage of caspase-1 are responsible for the processing of IL-1β and IL-18 precursors into biologically active cytokines. In turn, IL-1β induces IL-6 synthesis, as well as the production of IL-1Ra, a natural endogenous inhibitor of IL-1. IL-18, in combination with IL-12, is a major inducer of IFNγ production (37). Due to its extremely short half-life, IL-β is usually detected at very low concentrations in the bloodstream, even in disorders typically driven by IL-1β. As expected, we observed low serum IL-β concentrations in COVID-19 patients, which were, however, significantly higher than controls. Interestingly, we found high concentrations of IL-1β (reaching >10,000pg/ml in some individuals) in BALF, indicating intense activation of the pulmonary NLRP3-inflammasome and IL-1β production during COVID-19 disease. These observations corroborate and extend data from single-cell RNA transcriptomic analyses suggesting increased IL-1β in BALF of smaller cohorts of COVID-19 patients (38–40). In contrast to IL-1β, IL-1Ra and IL-6 are generally secreted at higher concentrations and appeared elevated in both BALF and serum of COVID-19 patients. In our study, serum IL-6 correlates with the severity of COVID-19, as previously shown by others (41, 42). Our results, showing abundant expression of activated caspase-1 associated with pulmonary vascular injury and thrombosed vessels, as well as elevated levels of NLRP3, caspase-1, IL-1β and IL-6 in the pulmonary microenvironment, confirm the role of the NLRP3-inflammasome pathway in COVID-19-associated immunothrombosis, as we previously suggested (14).

IL-18 was increased in BALF but especially in the serum of C-ARDS patients compared to controls, showing a positive association with disease severity as previously described (17, 43). However, serum IL-18 levels remained lower than those observed in other hyperinflammatory syndromes (44). We found low but detectable concentrations of IFNγ in C-ARDS patients, which in BALF appeared higher in C-ARDS and NC-ARDS than in controls. However, an IFNγ-induced signature is probably present in COVID-19, since CXCL10 (in common with CXCL9), an important IFNγ-induced chemokine, is highly detectable, as previously observed in several IFNγ-mediated syndromes (45). CXCL10 levels were elevated in BALF and serum of C-ARDS patients and correlated with disease severity. Several other reports have shown an elevated CXCL10 signature during COVID-19, including in BALF (39, 40, 46) and CXCL10 is a good circulating marker of disease severity (43, 47), consistent with the fact that a Th-1 signature can develop over time during severe COVID-19 (43, 48).

Inflammasome-dependent caspase-1 activation can also trigger pyroptosis with subsequent release of intracellular contents (49, 50). Increased levels of intracellular proteins such as NLRP3 and Casp1p20 observed in BALF and serum from C-ARDS patients suggest widespread cell death resulting from direct viral cytotoxicity or inflammatory damage. Consequently, we also found detectable levels of DAMPs such as IL-1α and HMGB1 in BAL of C-ARDS patients. These intranuclear proteins are released after cell death. They probably originate from injured cells of respiratory tractus, although the nature of these cells (bronchial epithelial, alveolar or endothelial…) remains uncertain. The specific contribution of DAMPs to COVID-19 immunopathogenesis remains incompletely understood, but the role of DAMPS is investigated in the review by Land et al. When viral load is too high, for example, in the respiratory tract, authors suppose a “forcing” of many virus-infected host cells to decide to commit “suicidal” regulated cell death (e.g., necroptosis, pyroptosis) associated with release of large amounts of DAMPs. Ironically, although the aim of this “suicidal” cell death is to save and restore organismal homeostasis, the intrinsic release of excessive amounts of DAMPs leads to those dysregulated hyperinflammatory responses—as typically involved in the pathogenesis of acute respiratory distress syndrome and systemic inflammatory response syndrome in respiratory viral infections (21). DAMPS could be released as a direct consequence of SARS-CoV-2 cytotoxicity, triggering cell necrosis, or indirectly because of the excessive inflammatory response, leading to necroptosis or pyroptosis associated with the release of large amounts of DAMPs. The excessive emission of DAMPS thus released into the extracellular environment could promote dysregulated hyperinflammatory responses, and pro-inflammatory programs, such as the activation of inflammasomes, notably the NLRP3 inflammasome (21, 51–53). In response to NLRP3 inflammasome activation, Caspase-1 is cleaved, resulting in pyroptosis. Moreover, IL-1ß and IL-18 released after cleavage by activated Caspase-1 belong to DAMPS. DAMPS could therefore participate in a positive feedback loop of the innate immune response induced by SARS-CoV-2 infection. The authors defined different classes of activating DAMPS in the event of cell lysis, and suppressor DAMPS or compensatory SAMPS in the initiation of controlled defense responses favoring, depending on their balance, either inflammation or resolution of inflammation in the event of infection (21). Two studies have reported elevated circulating concentrations of HMGB1 and calprotectin (S100A8/A9, a marker of neutrophil activation) in serum from COVID-19 patients (54, 55). In a study by Renieris et al., mice injected with serum from COVID-19 patients showed elevated pulmonary expression of pro-inflammatory molecules, including TNFα, IL-6 and CXCL10 resembling human COVID-19. Treatment of these mice with anakinra (which blocks both IL-1α and IL-1β signaling) or with antibodies selectively targeting IL-1α significantly attenuated COVID-19-like pulmonary immunopathology, potentially identifying IL-1α as a mediator of inflammation and tissue-specific injury (54, 56). Taken together, these observations may help to explain the limited efficacy of the anti-IL-1β monoclonal antibody, canakinumab, compared to anakinra in COVID-19 (57–61). Aware of the limitations of these observations, we plan to pursue this work by characterizing and specifically targeting DAMPS and eventually SAMPS in BALF and serum from ARDS patients.

We found that NLRP3-inflammasome activation and concentrations of IL-1α, IL-β, IL-6, IL-1Ra or CXCL10 were broadly similar in C-ARDS and NC-ARDS. However, concentrations of TNFα, sTNF-R1 and CXCL8 both in BALF and serum, were significantly higher in NC-ARDS than in C-ARDS, suggesting that inflammatory pathways involving NF-kB could be probably more up-regulated in NC-ARDS than in C-ARDS. A more likely explanation of these results may be due to the different use of steroids, which are well-known inhibitors of NF-kB-induced gene transcription. Glucocorticoids act by modulating the gene expression of several proteins involved in the inflammatory response. The reduced transcriptional activity of NF-κB is key to the anti-inflammatory effects of glucocorticoids. As expression of many of the pro-inflammatory cytokines implicated in COVID-19 is NF-κB-dependent, including IL-6, TNFα, and CXCL10 among others (62). Indeed, all C-ARDS subjects included in this study received steroids as a part of standard and recommended treatment, whereas NC-ARDS subjects were steroids free. It should be noted that all C-ARDS patients included in this study were in fact refractory to this steroid therapy, experiencing severe clinical deterioration despite well-conducted treatment, and that in these patients, steroids demonstrated almost a poor biological inhibitory effect on IL-1α, IL-β, IL-6, IL-1Ra or CXCL10 levels (62). The deterioration of COVID-19 patients is inextricably linked to immunopathological phenomena rather than viral load. The aggressive inflammatory responses in COVID-19 result in damage to the airways, termed ARDS, which may lead to respiratory failure and death. Corticosteroid treatment reduced mortality, but subsequent meta-analyses have again highlighted the importance of timing when considering corticosteroid administration, as corticosteroid use was associated with increased viral load. NLRP3 inflammasome activation is strongly correlated with COVID-19 severity and part of dexamethasone’s clinical effect in COVID-19 may be via NLRP3 inhibition (63). But our results suggest that these inflammatory cytokines,IL-1α, IL-β, IL-6, IL-1Ra or CXCL10, may be poorly targeted by steroids and may require to be specifically targeted in C-ARDS patients with poor response to steroids (64–66). Thus, in pursuit of this work, we aim to assess and analyze some of the anti-inflammatory cytokines, dependent from NLRP3 inflammasome pathway or from NF-κB pathway, to show how the balance between pro- and anti-inflammatory cytokines is affected in C-ARDS, NC-ARDS and control pulmonary vasculopathies, before and after steroid treatment. Furthermore, data showing similar IL-1/IL-6 cytokine profiles in C-ARDS and NC-ARDS patients suggest that these cytokine pathways may be highly activated in all types of ARDS, leading to the conclusion that ARDS should be considered as an hyperinflammatory state with DAD and severe prognosis in which novel anti-inflammatory strategies targeting these cytokines merit further mechanistic investigation (30). Specific NLRP3 inhibitors are currently undergoing clinical trials for the treatment of COVID-19 (63).

This study is limited in terms of power and clinical significance. Our control patients represented an imperfect control, since they were patients who might have incipient bronchopulmonary cancer. Bronchopulmonary cancer develops in inflammatory tumor microenvironment, which may involve the NLRP3 pathways. Tumor cell-intrinsic mechanisms of NLRP3 activation in the tumor microenvironment has been described by Tengesdal. et al (67). Aberrant activation of NLRP3 within the tumor microenvironment results in increased IL-1β and IL-18 secretion. Dysregulation of these cytokines induce tumor promoting mechanisms, such as angiogenesis, immunosuppression and metastasis. NLRP3 activation therefore represents a key immune checkpoint within the tumor microenvironnement, acting as a master switch for inflammation-mediated tumor progression. Nevertheless, according to our results from controls, ELISA protein expression levels of the inflammatory cytokines from the IL-1 family, and dependent from NLRP3 pathway, were significantly lower in BAL and in serum than in the other groups studied. Moreover, NLRP3 activation in cancer remains controversial. The role of the NLRP3 inflammasome therefore appears to be secondary in this inflammatory tumor microenvironment, after Transforming growth factor β (TGF-β). The article by Zhao et al. characterizes the tumor microenvironment in relation to carcinogenesis, and the predominant role of TGF-ß/SMAD4 signaling in cancer. TGF-β signaling pathway plays important roles in many biological processes, including cancer initiation and progression (68). Another predominantly inflammatory pathway, is interferon persistent signaling. Mathew et al. described the persistent IFN signaling as a potent immunoregulatory effect, promoting carcinogenesis. In cancer cells and immune cells, chronic IFN-I signaling is linked with cancer resistance in humans. IFN signaling thus appears to be involved in the carcinogenesis process, and argue to using combined JAK inhibition and PD-1 immunotherapy for non-small cell lung cancer patients (69). It seemed ethically difficult to propose another group controls totally asymptomatic.

Limitations of our study lie in the small number of patients in the study groups, and the missing data, resulting from mainly technical reasons, like insufficient quantity of samples or poor preservation of samples.

Finally, these results are therefore not generalizable but raise the hypothesis of an IL-1/IL-6 and IFNγ/IL-18 cytokine signature in ARDS BAL, in response to activation of the NLRP3 inflammasome in lung cells. These results are preliminary data to support clinical trials for new NLRP3 inhibitors, anti-IL-1 and anti-IL-6 therapies or JAK ½ inhibitors in COVID-ARDS and in ARDS.

As a conclusion, the results of this study support a role for the NLRP3-inflammasome activation and IL-1 in the immunopathogenesis of COVID-19. The presence of increased plasma levels of sNLRP3, sCasp1p20 and IL-1 cytokines (IL-1β, IL-18) was demonstrated in hospitalized patients across the spectrum of COVID-19 severity. Upregulation of this pro-inflammatory IL-1β/IL-6 pathway was also observed in both BALF and plasma obtained from critically ill patients with steroid-refractory-C-ARDS, but predominantly in BALF, better reflecting the inflammatory alveolar microevironment. Consistent with NLRP3-inflammasome activation, caspase-1 activation was detected on post-mortem lung tissues and mainly registered with lung-residing macrophages, localizing predominantly at the areas of alveolar injury and foremost-injured vasculature and thrombosis, hence supporting NLRP3-inflammasome activation as a putative mechanism contributing to immunothrombosis in COVID-19. IL-18/IFNγ-induced pathways also appeared to play a role in patients with steroid-resistant C-ARDS and may be insufficiently targeted by steroid therapy alone, arguing for new multi-target strategies in COVID-ARDS.

## Data Availability

The raw data supporting the conclusions of this article will be made available by the authors, without undue reservation.

## Supplementary material

Supplementary Figure 1 Flowchart of the study design including 3 cohorts of COVID-19 patients.

Supplementary Figure 2 Circulating IL-6, IL-1Ra and sNLRP3 concentrations correlated with clinical severity in C-ARDS. Correlations between circulating IL-1Ra **(A)**, IL-6 **(B)** and SOFA, or between soluble sNLRP3 and SAPS2 **(C)** in C-ARDS patients. SOFA, Sequential-Organ-Failure-Assessment; SAPS2, Simplified-Acute-Physiology-Score-2. Spearman R correlation coefficients are indicated.
