# Supplementary figures and images for: Caspase-1 activation, IL-1/IL-6 signature and IFNγ-induced chemokines in lungs of COVID-19 patients
Source: Front Immunol. 2025 Jan 15;15:1493306. doi: 10.3389/fimmu.2024.1493306 (PMC11774885; doi:10.3389/fimmu.2024.1493306)

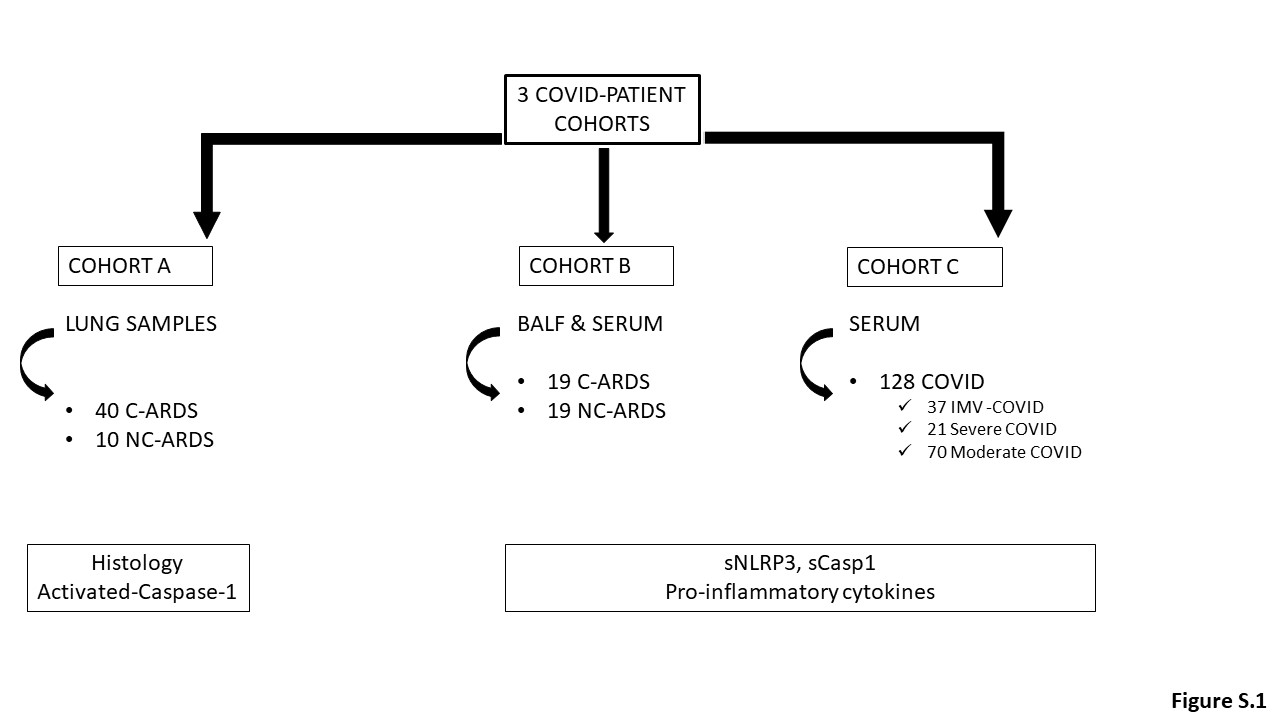

Supplement: Supplementary Figure 1 — Flowchart of the study design including 3 cohorts of COVID-19 patients. [file Image1.jpeg]

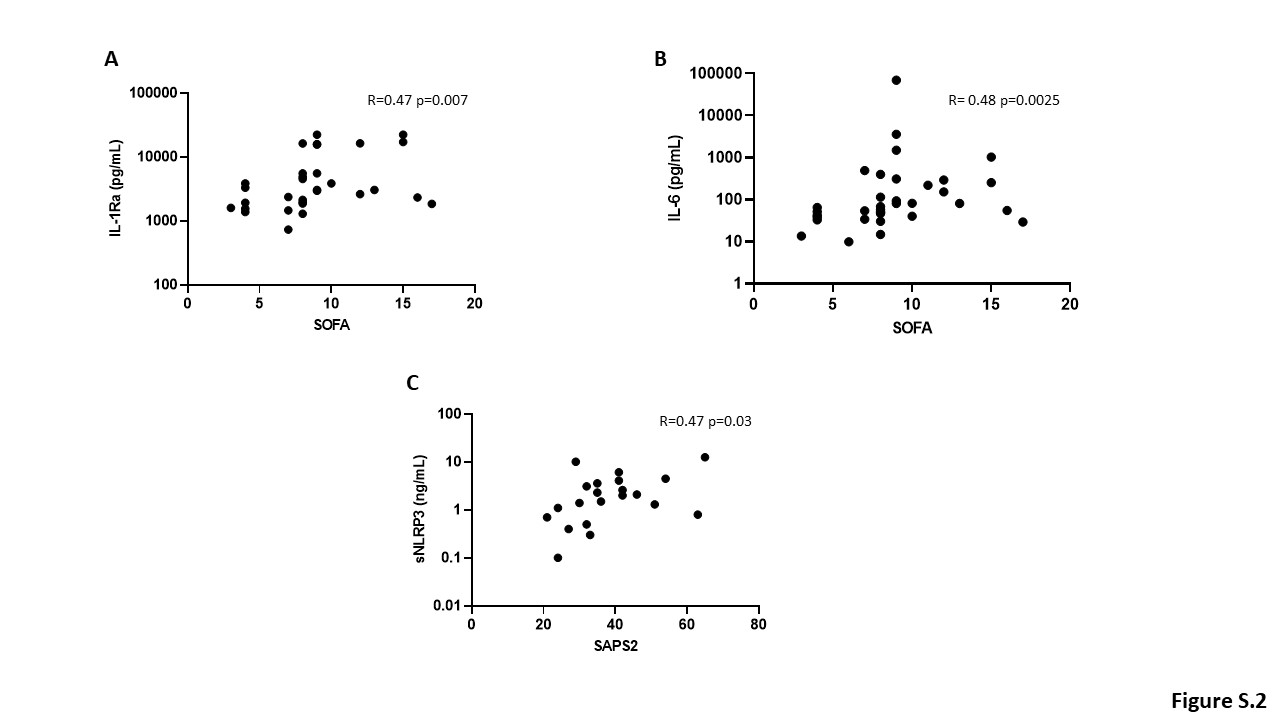

Supplement: Supplementary Figure 2 — Circulating IL-6, IL-1Ra and sNLRP3 concentrations correlated with clinical severity in C-ARDS. Correlations between circulating IL-1Ra (A), IL-6 (B) and SOFA, or between soluble sNLRP3 and SAPS2 (C) in C-ARDS patients. SOFA, Sequential-Organ-Failure-Assessment; SAPS2, Simplified-Acute-Physiology-Score-2. Spearman R correlation coefficients are indicated. [file Image2.jpeg]
